# Task Allocation of Wasps Governed by Common Stomach: A Model Based on Electric Circuits

**DOI:** 10.1371/journal.pone.0167041

**Published:** 2016-11-18

**Authors:** Allison Hilbun, Istvan Karsai

**Affiliations:** 1 Department of Biomedical Sciences, East Tennessee State University, Johnson City, Tennessee, United States of America; 2 Department of Biological Sciences, East Tennessee State University, Johnson City, Tennessee, United States of America; Universidade de Sao Paulo Faculdade de Filosofia Ciencias e Letras de Ribeirao Preto, BRAZIL

## Abstract

Simple regulatory mechanisms based on the idea of the saturable ‘common stomach’ can control the regulation of construction behavior and colony-level responses to environmental perturbations in *Metapolybia* wasp societies. We mapped the different task groups to mutual inductance electrical circuits and used Kirchoff’s basic voltage laws to build a model that uses master equations from physics, yet is able to provide strong predictions for this complex biological phenomenon. Similar to real colonies, independently of the initial conditions, the system shortly sets into an equilibrium, which provides optimal task allocation for a steady construction, depending on the influx of accessible water. The system is very flexible and in the case of perturbations, it reallocates its workforce and adapts to the new situation with different equilibrium levels. Similar to the finding of field studies, decreasing any task groups caused decrease of construction; increasing or decreasing water inflow stimulated or reduced the work of other task groups while triggering compensatory behavior in water foragers. We also showed that only well connected circuits are able to produce adequate construction and this agrees with the finding that this type of task partitioning only exists in larger colonies. Studying the buffer properties of the common stomach and its effect on the foragers revealed that it provides stronger negative feedback to the water foragers, while the connection between the pulp foragers and the common stomach has a strong fixed-point attractor, as evidenced by the dissipative trajectory.

## Introduction

Insect societies function as superorganisms [1] in which parallel processing is ubiquitous. The parallel processing not only makes the system more reliable [2], but it also makes possible the emergence of a complex system of the network of specialized units [3]. Division of labor is one of the most studied,debated, and intriguing phenomena in insect societies [4,5,6]. One of the most complex types of labor organization mechanisms is called task partitioning, which describes a situation when a given task, such as nest construction, is partitioned into subtasks. These subtasks are commonly connected sequentially and carried out by different more or less specialized individuals, such that it can be observed on the working process of the bucket brigade [7]. The assignment of a given worker to a given subtask is commonly dynamic, because it depends on the progress of the work, the number of participants, and other factors, and it poses a decision problem at the individual level for task switching [8]. In the insect society, each agent has only a local perception and only local information about the overall situation, and these societies have no foreman or other central task allocation unit, therefore the whole system is self-organizing itself to establish efficient performance via allocating different numbers of workers to different task groups [9,10,1,11].

Swarm founding *Metapolybia* wasps exhibit flexible and adaptive task specialization, in which distinct subsets of the complex nest construction task is partitioned between cooperative teams of nest mates [3,12,13]. The building task is partitioned into four subtasks, and all subtasks are carried out by generally different individuals. Some workers specialize in water collecting and bring the water to the nest, where it is stored in the crop of other wasps. These water storer wasps form a “common stomach” where the water can be downloaded or taken out, if needed. Other specialized wasps called pulp foragers collect water from the common stomach and fly out to collect wooden pulp. The water they bring from the nest is needed to macerate the plant materials (cellulose) into building material. This building material then is transported to the nest, where it will be distributed to builder wasps, which built the pulp into the nest. Field experiments and modeling of this system revealed that the saturation of the common stomach is used by the wasp as an information center [14]. For example, if the common stomach is saturated with water, the water foragers have difficulty downloading their water load, while the pulp foragers can take water from the common stomach very easily. This indicates that in the colony, there would be more water providers than necessary. Consequently, some of the water foragers would give up water foraging and switch into water users such as pulp foragers or builders. However, these switches also have costs [15]; therefore a large common stomach also can play a role as a buffer [16], so small fluctuations would not trigger task switching, and the wasps would operate with high task fidelity [17]. This would in turn ensure additional benefits to the colony, such as the ability to learn the position of water and pulp resources.

Task partitioning itself is an old and general challenge not only in insect societies [18–20], but also in computational distributed systems [21–22] or in robot groups [23–24]. Due to the hiatus of master equations in biology, task partitioning is commonly described and modeled with agent-based approaches or by the use of empirical functions. For example the ‘‘response threshold models” assume that workers vary intrinsically in task preference [25] and these threshold functions are commonly described by some form of sigmoid curve [26]. Karsai and Balazsi [27] used a Weibull function, commonly used to describe stress and aging processes, for modeling task partitions and Karsai and Schmickl [14] built a complex system dynamic model that used combinations of linear functions to describe material flow and task switching in *Metapolybia* societies. These models are based on empirical data, fitted functions, and simple reasonable assumptions which well predicted the operation of natural colonies.

Our goal in this paper is different. We will show that the essence of this complex biological phenomenon can be described by master equations using the physical systems of inductance circuits. We have built a model from electric circuits that will provide similar predictions to that which we observed in real colonies and empirical models. Models based upon electrical circuits have been adeptly used to model such systems as the nervous system; Hodgdon and Huxley [28] provided a circuit model, based upon resistors and a capacitor, to model nerve impulses. Their research has been confirmed and expounded upon for further elucidation of cellular processes such as anesthesia [29]. Furthermore, it has been asserted that the properties of neural circuits and animal behavior are linked [30]. Coupled circuits involving capacitors, resistors, and inductors were chosen for this model due to the circuits’ inherent abilities to essentially explain storage through inductors and capacitors, loss due to environmental factors through resistors, and a general structure that would allow for a circular flow of a supply of particles: water in the biological system and electrons in the physical circuit. We will carry out a series of perturbation experiments in our model and we will compare the predictions or our model to field experiments and the predictions of other models constructed for the same system.

## Methods

### Theory/Calculations

**Fig 1:**
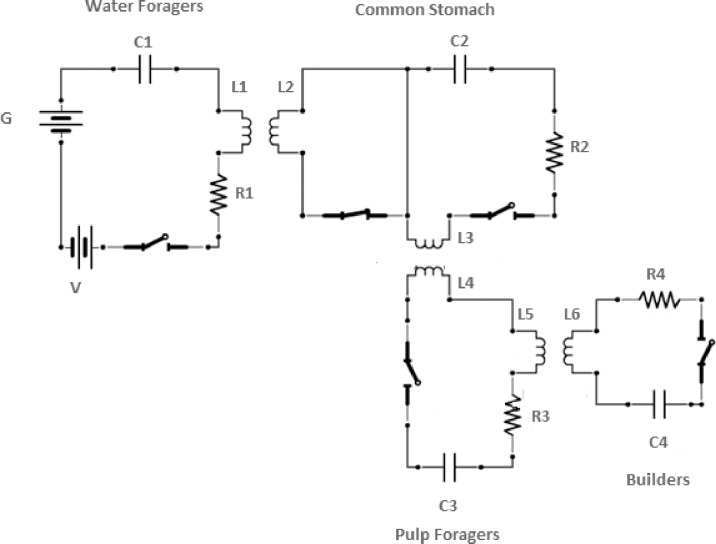
Circuit diagram model of task partitioning of *Metapolybia* wasps. The four circuits represent the group of wasps belonging to the four task groups. Elements of circuits are described in Table 1.

Our model is based upon a simple physical system: an electrical circuit. It consists of four circuits, each of which acts as a different functional part of the wasp colony. The four parts of the wasp system which are modeled are the water foragers, the common stomach, the pulp foragers, and the builders. The electrons flowing through the circuits are used to model water flow through the system. In other words, each circuit corresponds to a group of wasps that are engaged in the same task, and the flow of electrons represents the flow of water through the system. Each task group (water foragers, pulp foragers, common stomach wasps, and builders) are represented by an RLC circuit. These circuits are related to simple harmonic oscillators. The inductance (L) acts as the mass of a harmonic oscillator system; the resistance (R) causes damping, and the capacitance (C) behaves like the spring constant of a mass oscillating on a spring. Each of the individual RLC circuits is connected by mutual inductance, representing the connectedness of these tasks (Fig 1). Mutual inductance was chosen as a connection between the circuits because the change of mutual inductance in one coil induces a current in the second coil. The water foragers acquire water, and then the water foragers directly affect the common stomach, the common stomach directly affects the pulp foragers, and the pulp foragers directly affect the builders. Because the water foragers collect water and increase the supply of the common stomach, we use mutual inductance to model this work-based exchange of materials and information. Because in order to forage pulp, the pulp foragers need water from the common stomach, there is once again a work-based exchange of materials and information. The builders need supplies from the pulp foragers to build, so these circuits are also connected by mutual inductance. Sinks of water for construction and drinking/cooling are modeled via resistors.

The current model is different from previously published models of task allocation of wasp societies [14,15,16,17,27], because these models used either an agent-based approach or a combination of empirical and linear equations. Our present electrical circuit approach models the flow of water through the wasp system using master equations, which are based upon a well-studied physical system [31]. This new model also allows us to derive new testable predictions on the connectedness of the system as a whole and the connectedness of the common stomach with the pulp foragers.

### The Model

#### The Water Foragers

The task of the water foragers is to collect water and transfer the water to the common stomach. The circuit which models the water foragers comprises a capacitor (C1), a resistor (R1), and an inductor (L1). This circuit also has two voltage sources, (V and G), allowing current to flow in two different directions and for directionality to be adjusted. The water forager circuit’s inductor (L1) is placed in close proximity to the inductor (L2) of the common stomach to allow for flow between the circuits. The work of the water foragers are regulated by how full the common stomach is and how much water is generated by the main water source (G).

#### The Common Stomach

The common stomach RLC circuit is comprised of a resistor (R2), a capacitor (C2) and two inductors (L2 and L3). The function of the common stomach is such that water foragers can download water into it and the pulp foragers can upload water from it. This flow of materials is modeled by L1 and L2 as well as L3 and L4 inductors being placed in close proximity (Fig 1). The L1 from the water foragers allows electrons to flow through the common stomach, and the L3 from the common stomach allows electrons to flow to the pulp foragers. There is a wire which divides the circuit so that this circuit will have two switches, each affecting a different inductor, allowing for feedback. One switch opens when the other switch closes, and vice versa. This causes changes in the magnetic field so that voltage can be induced in adjacent circuits (Faraday’s Law). Additionally, because the common stomach is a temporal storage place, it has a high capacitance capability (Table 1). The resistor (R2) of the common stomach reflects the common stomach’s potential use of the water for other reasons than construction, such as consumption and cooling. It has been shown that as the common stomach saturates, it decreases the flow into the common stomach, which means that the percentage of water foraging has decreased [14]. We modeled this property by simply placing the common stomach near both the inductor coils of the water foragers and the pulp foragers. Its central location here allows it to act as a buffer and also provide feedback to the system.

**Table 1:** Parameters of the Model.

**Parameter/variable**	Description	Value/unit
C1	Capacitor WF	0.5 F
C2	Capacitor CS	10 F
C3	Capacitor PF	3 F
C4	Capacitor B	10 F
R1	Resistor WF	5 Ω
R2	Resistor CS	5 Ω
R3	Resistor PF	5 Ω
R4	Resistor B	10 Ω
G	WF Battery part 1	Sin[1.5t]*e^-t^ V
V	WF Battery part 2	1 V
L1	Inductance WF	5 H
L2	Inductance CS + WF	10 H
L3	Inductance CS + PF	5 H
L4	Inductance PF+CS	5 H
L5	Inductance PF+B	5 H
L6	Inductance B	5 H
M1_a_	Mutual Inductance WF→CS	0.1 H
M1_b_	Mutual Inductance CS→WF	0.2 H
M2	Mutual Inductance CS↔PF, PF↔B	0.1 H

* WF = water foragers, CS = common stomach, PF = pulp foragers, B = builders

#### The Pulp Foragers

The pulp forager RLC circuit is a combination of a capacitor (C3), resistor (R3), and two inductors (L4 and L5) in series, and it is connected to the common stomach via the inductor L4 and it is connected to the builders via L5 (Fig 1). To simplify the system, we assumed that the pulp foragers simply convert water to watery pulp, therefore the water is lost only in small quantities through R3 (some water evaporates during pulp making).

#### The Builders

The builders’ RLC circuit is a combination of a capacitor (C4), resistor (R4), and an inductor (L6) in series, and it is connected to the pulp foragers via the inductor L5 (Fig 1). The resistor (R4) in this circuit drains the circuit of electrons and this loss of energy from the system represents the wasp building process, where the water is in the form of pliable building material, which will dry out after the construction finished.

### General Assumptions

In our model, electron flux is representative of water flow. Wasps belonging to the same task are grouped into a single circuit. The main source of electrons to the system is originated from the batteries of the water forager circuit (G and V). The wires in the system do not allow for dissipation of energy; the wires are completely efficient, as decrease of current is only supposed to occur at the resistors. There are no time delays in the circuit wires. Additionally, it is assumed that the changes in current causing voltage to be induced are equal to the charge on the adjacent circuit. The second derivative term, representing the voltage which is induced from one coil to the next, is thus equivalent to the charge of the adjacent circuit in this system. When perturbation experiments were carried out, if something was removed from the system, we assumed that this quantity was not replaced; therefore the system reached a new equilibrium based on the changes. The switches in the system can be opened and closed at appropriate times in order to cause a change in magnetic field and subsequently create a voltage in the proximal coil. Additionally, all switches in the system are assumed to open and close in such a way as to allow continuous electron flow between circuits and thus the circuit is assumed to allow for both the transfer of electrons through the inductors and also return to a state of equilibrium.

In the circuit models, the electrons are generated by batteries, while in the wasp colonies, the water is collected by water foragers. This difference between the two systems is especially important for studying the effect of perturbations on the water foragers. In wasp colonies, the effect of a perturbation is commonly propagated through the whole system, but the continuous generation of electrons in a water forager circuit could flood the water forager circuit with electrons, therefore the backpropagation of the perturbation could not be detected easily. To keep the model simple, but make the effect of perturbation detectable, the mutual inductance between the common stomach and water foragers (M1) has 2 different values (either M1_a_ or M1_b_, depending upon the origin of the current change). M1 = M1_a_ when current change originates within the water foragers and M1 = M1_b_ when current change originates within the common stomach (or the pulp foragers or builders which in turn change the current in the common stomach). We assumed that the effect of common stomach to the water foragers is larger than the opposite effect, hence M1_a_ < M1_b_ (Table 1). This could be conceptualized as a step up transformer and this setup improves the detection of the effect of perturbations to the water forager circuit.

The parameters of this model were not possible to obtain directly from the biological system, but we parameterize this model to adhere the biological system as closely as possible. We also follow the simplicity principle and therefore, if there is no indication in the biological system that similar parameters should be markedly different (for example water use for drinking of different types of wasps (R1-R3)) then we use the same values for the resistors except for R4, which also represents the water loss via the evaporation of water from the freshly constructed structure (Table 1). Generation of water is assumed to have a steady (V) and a fluctuating (G) component, which was described by a simple sinus function. The values of capacitors are different, because it represents the size of the task group of the wasps in the colony. The colonies generally operate only with few water foragers, more pulp foragers and larger number of builders and common stomach wasps [26]. We used inductance values to fine tune the basic model to predict realistic ratios between the task groups (Table 1).

### Behavior of the 4 circuits

Our model consists of four RLC circuits coupled by mutual inductance, simulating the wasp colony’s water and pulp foraging, the operation of the common stomach, and the building. The different behavior of the 4 tasks (change of charge on the 4 loops), is solved by Kirchoff’s basic voltage laws. The four loops are described separately by simple second order differential equations (Eqs 1–4) to study the responses of the tasks independently; P, W, C, and B refer to the charge on each RLC circuit for the pulp forager, water foragers, the common stomach, and the builders, respectively.

The change of charge in time in the water forager circuit is described by

$$W^{\prime \prime }[t]=\frac{\left(C^{\prime \prime }[t]*M1-R1*W^{\prime }[t]-(\frac{W[t]}{C1})+V_{Battery}\right)}{L1}$$ (Equation 1)

Where the *C*″[*t*] * *M*1 term represents the mutual inductance term of the water foragers connected with the common stomach, and M1 = M1_a_ with current change originating in the water foragers and M1 = M1_b_ for current change originating from the common stomach; R1*W[t] is the voltage drop due to the resistor representing the water use of the water foragers. W[t]/C1 is subtracted as the voltage drop across the capacitor, showing the water foragers’ ability to retain small quantities of water. The water inflow is modeled via the battery voltages, V and G (Fig 1). These are summed and are referred to as V_Battery_. The right hand side of the equation is divided by L1, which was derived as *W*″[*t*] multiplied by L1 as the change of current times the inductance of this circuit, also caused by mutual inductance. The equation is set equal to zero, and then solved for W″[*t*], causing all terms to be divided by the L1 inductor.

The change of charge in time in the common stomach circuit is described by

$$C^{\prime \prime }[t]=\frac{\left(W^{\prime \prime }[t]*M1-P^{\prime \prime }[t]*M2-R2*C^{\prime }[t]-(\frac{C[t]}{C2})\right)}{L2+L3}$$ (Equation 2)

*W*″[*t*] * *M*1 represents the first mutual inductance term of the water foragers acting with the common stomach, and M1 = M1_a_ with current change originating in the water foragers and M1 = M1_b_ for current change originating from the common stomach; *P*″[*t*] * *M*2 represents the second mutual inductance term of the common stomach creating mutual inductance with the pulp foragers. These two terms allow the transfer of water to the common stomach by water foragers and from the common stomach by the pulp foragers. The *R*2 * C′[*t*] term is subtracted as the voltage drop across this resistor, showing the small loss of water from the common stomach. The $\frac{C[t]}{C2}$ term is subtracted for the capacitor; this is a large capacitor, because the common stomach plays the role of water storage, buffer and eventually regulating the wasp activity. The nominator on the right hand side of the equation is divided by L2 + L3, because the derivation was that *C*″[*t*] * (*L*2 + *L*3) represents the second aspect of the mutual inductance, which is dependent upon the inductance of the individual coils that are in the common stomach coil. The equation is solved for *C*″[*t*], so the right hand side of the equation is divided by L2 + L3.

The change of charge in time in the pulp forager circuit is described by:

$$P^{\prime \prime }[t]=\frac{\left(C^{\prime \prime }[t]*M2-B^{\prime \prime }[t]*M2-R3*P^{\prime }[t]-(\frac{P[t]}{C3})\right)}{L4+L5}$$ (Equation 3)

where *C*″[*t*] * *M*2 represents the mutual inductance term of the pulp foragers connected with the common stomach and *B*″[*t*] * *M*2 represents the mutual inductance term of the pulp foragers with the builders. These two terms allow the transfer of water from the common stomach to the pulp foragers and from the pulp foragers to the builders. *R*3 * P′[*t*] is simply the voltage drop across the resistor from Ohm’s Law, showing water use (other than pulp collecting behavior) of the pulp foragers. P[t]/C3 is subtracted as the voltage drop across the capacitor, showing the pulp foragers’ ability to store small quantities of water. The nominator of the right hand side of the equation is divided by L4 + L5, which was derived as the change of current times the inductance of this circuit, also caused by mutual inductance. The final equation shown above is set equal to *P*″[*t*], so the right hand side is all divided by L4 + L5, the inductance of the pulp forager coil and thus its ability to accept water from the common stomach.

The change of charge in time in the builder circuit is described by

$$B^{\prime \prime }[t]=\frac{\left(P^{\prime \prime }[t]*M2-R4*B^{\prime }[t]-(\frac{B[t]}{C4})\right)}{L6}$$ (Equation 4)

*P*″[*t*] * *M*2 represents the first mutual inductance term of the pulp foragers acting with the builders. This models the water arriving to the builders as a wet pulp. The *R*4 * B′[*t*] term is subtracted as the voltage drop across this resistor, modeling the evaporating water leaving the freshly constructed nest material. The $\frac{B[t]}{C4}$ term is subtracted for the capacitor showing the builders’ ability to store small quantities of water. The numerator on the right hand side of the equation is divided by L6, because the derivation was that *B*″[*t*] * *L*6 represents the second aspect of the mutual inductance, which is dependent upon the inductance of the individual coils that are in the common stomach coil. The equation is solved for *B*″[*t*], so the right hand side of the equation is divided by L6.

## Results

### General behavior of the system

The circuit model produced plausible predictions for the colony level behavior of wasp societies [32] and comparable results to the earlier empirical models [14,27].

**Fig 2:**
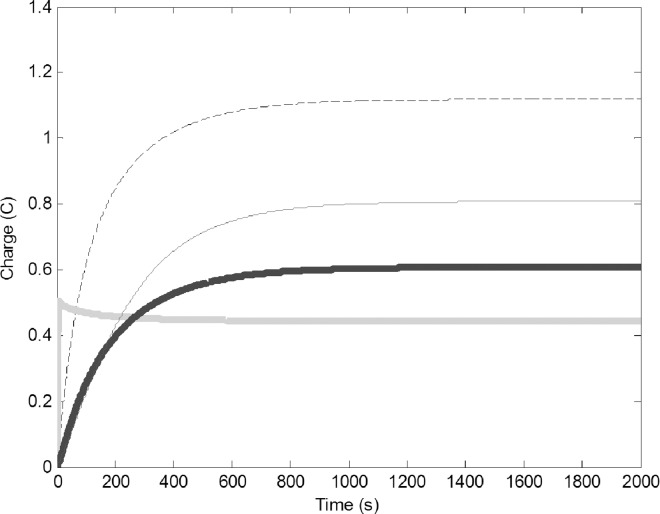
Change of charge on the four circuits after the batteries V and G are turned on (t = 0). Common stomach: dashed line, builders: thin black line, water foragers: thick gray line, pulp foragers: thick black line. The model used the basic parameters (Table 1).

Assuming zero initial charge on all circuits, there was a sharp increase of charge in the water forager circuit at the very beginning of the simulation, which quickly reached equilibrium. This was followed by an increase of charge in the common stomach circuit. Finally, the pulp foragers and the builder circuits increased in a delayed manner and reached equilibrium at approximately 1200 seconds (Fig 2), when the electrons generated by the battery propagate through the system, damped by the resistors. Overall, the model predicted a continuous construction where the charge of PF was larger than of WF, similar to wasp colonies, where there are more pulp foragers than water foragers. The charge on the builders is higher than that of the foragers, which was also found in actual colonies, where more builders exist than foragers. The common stomach has a higher charge than the foragers and this in fact is important to ensure the buffering ability of the common stomach. The values at which these circuits stabilize are independent of the initial charge on the circuits.

### Perturbation Experiments

In order to further test the robustness of the model, perturbation experiments were carried out and the model predictions were compared to field data and the predictions of previous models, qualitatively. Removing or adding components of the system or materials will force the system to adapt. For example, spraying water on wasp nests increases construction, because the water foragers can get water quickly on site, which in turn increases the water level of the common stomach. This will promote more pulp foraging; with more pulp arrives to the nest with a higher rate, more construction will result [32].

All simulations started as the normal run, but after the system stabilized at time t = 2000 seconds we made a sudden change in a single parameter and followed the change of the charge of the four circuits (the water foragers, the pulp foragers, the common stomach, and the builders). The direction of the change of different circuits is compared to the observed change in the number of pulp and water foragers [32].

**Fig 3:**
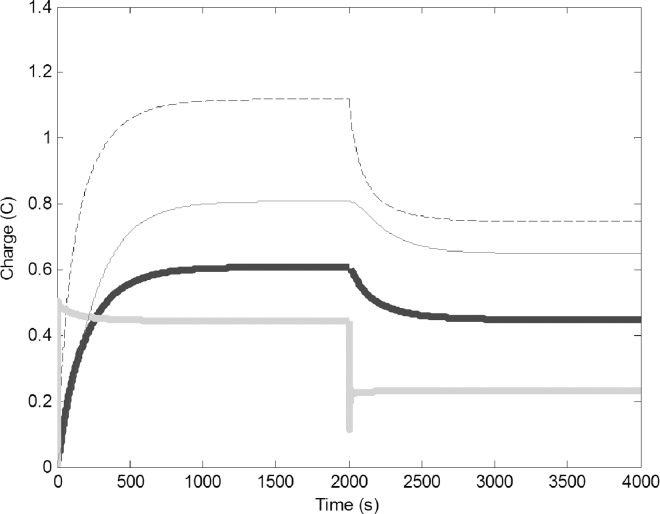
Removal of water foragers (decrease of C1 from 0.5 to 0.25 F and R1 from 5 to 2 Ω); Common stomach: dashed line, builders: thin black line, water foragers: thick gray line, pulp foragers: thick black line.

To simulate capturing water foragers (removing members of this task group), we suddenly decreased their number by reducing C1 from 0.5 F to 0.25 F and reducing R1 from 2 to 1 Ω (Fig 3). The reduced number of water forager was unable to refill the common stomach, this in turn resulted in reduction of charge on all circuits. There is a concurrent drop of reserve water in the common stomach, which decreases the number of pulp foragers that can use the water to forage. New equilibriums were established, accommodating the lower electron flow.

**Fig 4:**
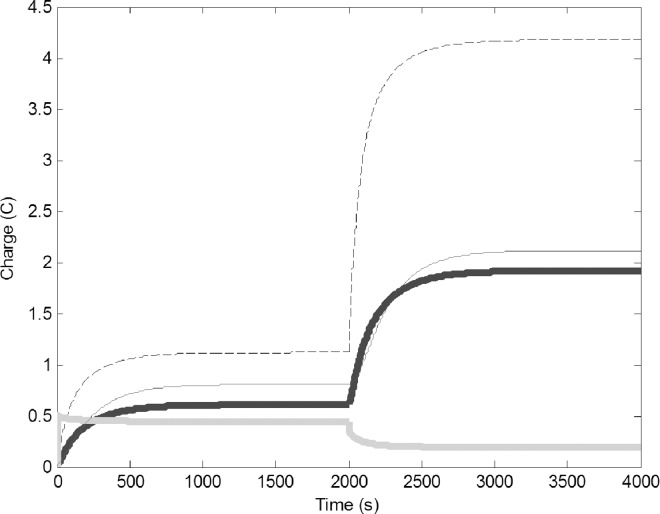
Addition of water to the environment (0.5 V added to G, increase of water output from the environment): Common stomach: dashed line, builders: thin black line, water foragers: thick gray line, pulp foragers: thick black line.

To simulate addition of extra water to the environment, we increased the water output by adding 0.5 V to the fluctuating battery component (G). Because of the direction of the wires, increasing G would result in a decrease in the charge on the water foragers, but the change in current should induce voltage in the other circuits (Fig 4). The charge on the water foragers decreased and the charge on the common stomach increased. This in turn increased the charge of the pulp forager and builder circuits. This behavior is very similar what we can observe in wasp colonies after the rain. Increased water availability will make the refilling of the common stomach easy, therefore part of the water foragers are converted to pulp foragers and builders.

**Fig 5:**
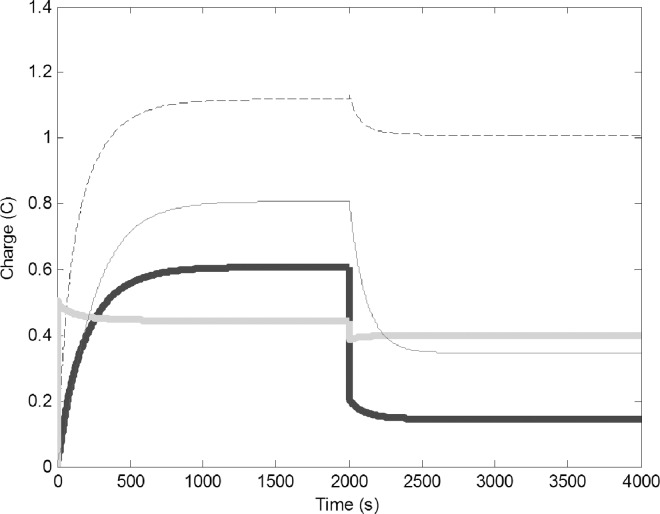
Change in time of charge when pulp foragers are removed (C3 reduced from 3 to 1 F and R3 reduced from 5 to 2 Ω). Common stomach: dashed line, builders: thin black line, water foragers: thick gray line, pulp foragers: thick black line.

Modeling the removal of pulp foragers was carried out by assuming the physical circuit decreased proportionally in size, causing a decrease in C3 from 3 to 1 F and a decrease of R3 from 5 to 2 Ω. Fewer pulp foragers collected less pulp, therefore the colony had needed less water, so both the number of builders and water foragers decreased. The amount of water in the common stomach decreased as well (Fig 5).

**Fig 6:**
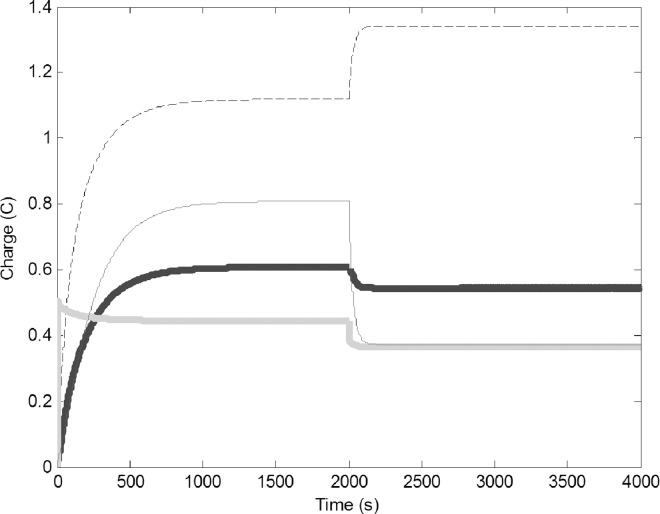
Change of charge in time when builders are removed (C4 is reduced from 10 to 5 F, and R4 is reduced from 10 to 5 Ω). Common stomach: dashed line, builders: thin black line, water foragers: thick gray line, pulp foragers: thick black line.Leakage of the common stomach was modeled by the drain of 0.05 V from the location of the resistor R3 in the common stomach (Fig 7). This caused a significant decline in the charge on the Common Stomach, and this in turn decreased pulp foraging and building. The number of water foragers increased as a compensatory effect for increasing water influx into the system.

Decreasing the number of builders was modeled by assuming the physical circuit decreased proportionally in size, causing a decrease of C4 from 10 to 5 F, and a decrease of R4 from 10 to 5 Ω. (Fig 6). Decreasing building capacity decreased the demand of pulp and in turn the water, which would be why the number of foragers dropped. Due to less water use, the water in the common stomach increased, but the influx and outflux of water into the common stomach became much slower.

**Fig 7:**
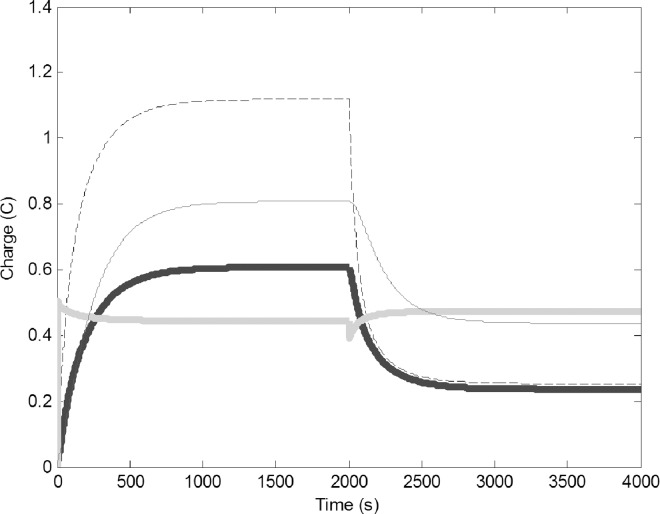
Change of charge in time implementing a leaky common stomach (0.05 V drained from R3): Common stomach: dashed line, builders: thin black line, water foragers: thick gray line, pulp foragers: thick black line.

**Fig 8:**
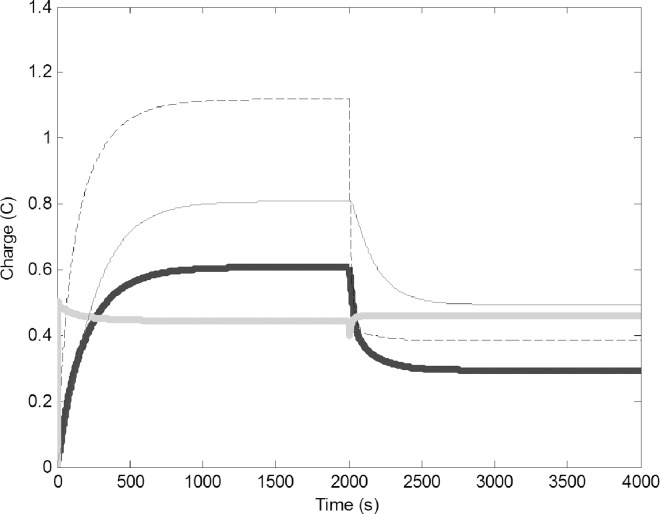
Change in charge of circuits when the storage capacity of the common stomach is reduced (C2 reduced from 10 F to 3 F.): Common stomach: dashed line, builders: thin black line, water foragers: thick gray line, pulp foragers: thick black line.

To simulate change in the capacity of the common stomach we reduced C2 from 10 to 3 F. This would mean that the common stomach wasps did not have their full ability to store water or the number of water storer wasps decreased. Since they were not capable of storing adequate amounts of water, the water foragers were forced to increase to attempt to sustain the demand for water for the builders and pulp foragers (Fig 8). However even with larger water foraging the pulp foraging and building decreased into a lower equilibrium.

**Fig 9:**
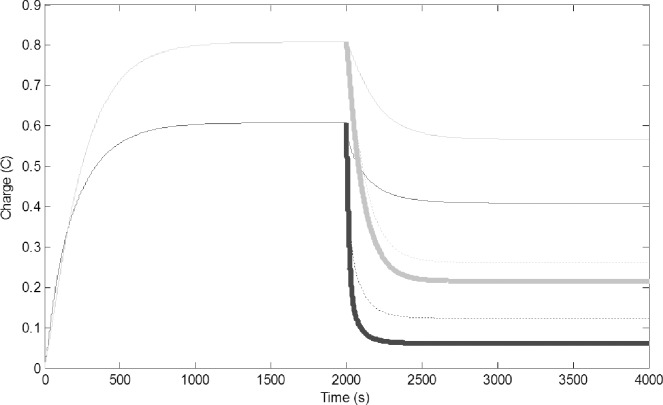
Change of charge in time with different coupling intensity between the circuits (M1 and M2 were reduced from 0.1 H to 0.01 H (thickest line), from 0.1 H to 0.05 H (dot/dashed line), and from 0.1 H to 0.75 H (medium thick line); pulp foragers (black), builders (gray). With decreased coupling, the charge on the circuits drops due to decreased current passed through each circuit by the inductors.

Mutual inductance (M1 and M2) of the system, which serve as the linkage between the task groups, were manipulated to test the reliance of the common stomach and the pulp foragers on the water foragers. M1 and M2 were reduced from 0.1 H to 0.075 H, 0.05 H, and 0.01 H, to represent a 25%, 50%, and 90% reduction, respectively. This resulted in the plummeting of the charge on the pulp foragers and builders (Fig 9). Our standard simulations had high mutual inductance between the circuits, because the wasp society we studied is highly connected. Decreasing the mutual inductance means that the task groups became less coupled. This resulted in large drop in the charge of all circuits indicating that this system is not effective with low linkage. Decreasing the inductance simulates the situations where the task groups are loosely connected and the society operates with less specialized individuals [3].

**Fig 10:**
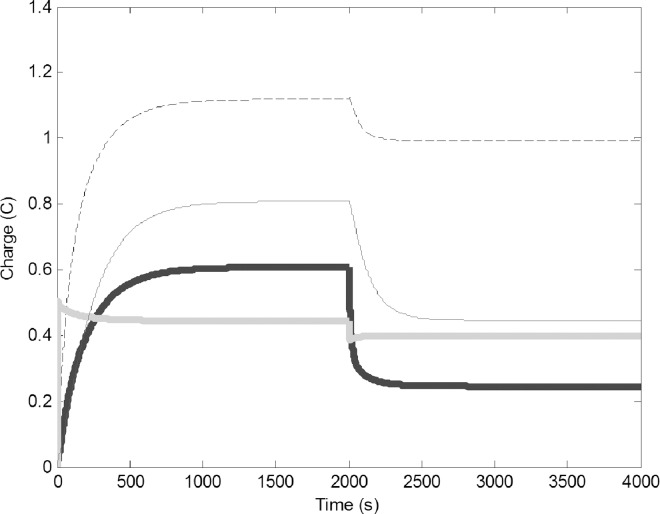
Change in charge of circuits when the connectivity between the common stomach and the pulp foragers is reduced (mutual inductance M2 between common stomach and pulp foragers reduced from 0.1 to 0.05 H). Common stomach: dashed line, builders: thin black line, water foragers: thick gray line, pulp foragers: thick black line.

Simulating difficulty to obtain water from the common stomach by the pulp foragers indicates a situation when conserving water is important to the colony. This was achieved by decreasing the coupling between the two circuits by decreasing the mutual inductance between the common stomach and the pulp foragers (M2) from 0.1 to 0.05 H. This resulted in the significant decline in charge of the common stomach, foragers, and the builders (Fig 10). This reflects the difficulty in sustaining pulp foraging and thus building if the transfer of water is hindered between the common stomach and the pulp foragers.

**Fig 11:**
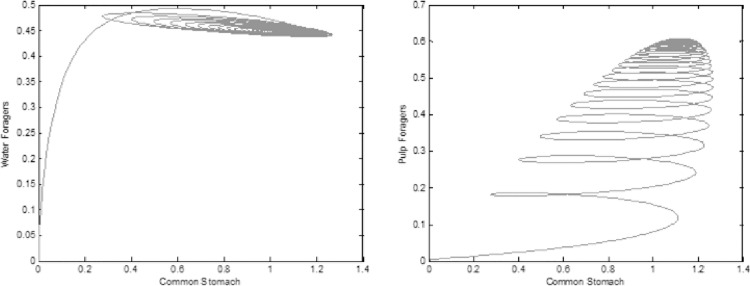
Phase plots of charge of foragers vs. the common stomach. a: Common stomach vs. water foragers with low common stomach resistance (R1 reduced from 5 to 0.000001 Ω). b: Common stomach vs. pulp foragers with low common stomach resistance (R2 reduced from 5 to 0.000001 Ω), The start point is at the origin.

To demonstrate that the common stomach has different types of relationships with the foragers, the charge of the common stomach was plotted vs. the charge of the foragers. In these experiments, we assumed low water consumption (R2 reduced from 5 Ω to 0.000001 Ω), (Fig 11). The phase-space plots show that the number of water foragers quickly stabilize, while the common stomach oscillated in a damped fashion. The pulp foragers’ relationship with the common stomach is different from the water foragers’, because their charge oscillates longer following the oscillations of the common stomach and they together slowly decay toward a stable point. This suggests that the common stomach’s relationship with the water foragers involves more feedback than the relationship between the common stomach and the pulp foragers.

## Discussion

The common stomach as a regulatory mechanism for task partitioning and work allocation has been shown in wasps [15,17,27,32,3], ants [33] and bees [34]. These agent-based or system dynamic models are based on empirical functions and observations and provide numerically calculated predictions for comparison with empirical data and other models. Our goal in this paper was to apply master equations of circuit dynamics to describe our biological system and to show that the circuit model we present here is not only able to provide similar predictions to empirical models, but it is also able to explore new relationships between variables, and hence can promote new experimentation.

Our circuit system is very minimalistic, but it is capable of modeling the task allocation of the social wasps and the predictions of the model are very similar to what we can observe in the field or using empirical models [17,27,32]. The system is strongly controlled by the common stomach, which is akin in several ways to the information center introduced by Seeley [35] in bees. The water providers and users are interacting indirectly through the common stomach, which not only provides information on the status of water flow, but also buffers the system. The system works independently of the initial conditions shortly set into an equilibrium, which provides optimal work/task allocation for a steady construction, depending on the influx of accessible water. The system is very flexible and in the case of perturbations, it reallocates its workforce and adapts to the new situation with a different equilibrium level.

This flexibility of task reallocation correlates with other life-history parameters, such as colony size, body size, and nesting habits [3]. The wasp colonies we modeled here are of medium size, and individual workers are not strongly fixed in a task. The individuals can change their behavioral profile quickly [32], therefore we assumed that every worker wasp is identical to the others and they differ only in which task group to which they belong. This allowed us to simplify this system into group levels and describe the groups as circuits. Our model has intrinsic differences between the parameters of the circuits, but each circuit is intrinsically the same type of circuit (RLC).

Perturbation of the circuit model predicted changes that similar to the field observation on *Metapolybia* [27] and *Polybia* [36] wasps. Addition of water to our system increased pulp foraging and construction, but decreased water foraging. The removal of water foragers also decreased the charge on all involved circuits, meaning that the system was set to a lower equilibrium until new wasps could be recruited for water foraging. Decreasing pulp foragers showed a decrease in charge to all circuits except for a slight increase in charge for the common stomach, due to its central position and enhanced storage capacity. The circuit model was able to predict all major perturbations qualitatively the same manner as it was observed in the field and in other models [15,27,32,3].

Karsai and Wenzel [3] analyzed several life history parameters of many wasp species and their main finding was that simple individual level behaviors and interactions will lead to variances in life history, such as how flexible the behavioral repertoire of the individual is and how connected the subsystems are via interactions. The two extremes of this scale are the small societies with independently acting jack-of-all-trade individuals and the strongly connected more rigid behavioral or age based caste systems of large colonies. Our model, via manipulating the mutual inductance terms and the storage capacity of the common stomach, allowed us to predict what would happen if these interactions (or the connectedness of the circuits) are weakened. The model predicted a large decrease of construction related activities including drop of water content in the common stomach. In fact, the nest construction based on common stomach is not viable in societies with small number of wasps. *Metapolybia* and *Polybia* wasps are breeding via colony fission, therefore their colony size normally does not go too low [37]. Wasps with small societies are using jack-of-all-trade workers and they do not have common stomachs [3].

Our model also was able to explain the differences regarding how water foragers and pulp foragers connect to the common stomach. Pulp foragers generally spend more time and a higher number of interactions with common stomach wasps than water foragers do [32]. Our model predicted different dynamics between the two forager types with the common stomach. In the phase-space plots, the relationship between the foragers and the common stomach both have point attractors, however, the water foragers observably orbit around the common point attractor and quickly reach a close spot to that attractor, but then spirals away from it (Fig 11A). The phase space plot of pulp foragers vs. the common stomach reveals a consistent dissipation towards the point attractor (Fig 11B). This suggests that the common stomach’s relationship with the water foragers involves more negative feedback than the relationship between the common stomach and the pulp foragers. We propose that the task allocation via the common stomach is a very efficient regulatory mechanism, because through a network of worker interactions, a set of positive and negative feedbacks are connected and balanced by a robust buffer system.
